# The Trilemma of Today’s Aging Population in the Time of Pandemic: A Case Study of Pre-existing Psychiatric Illness and Cognitive Deficits, COVID-19, and Further Cognitive Decline

**DOI:** 10.7759/cureus.28725

**Published:** 2022-09-03

**Authors:** Mack E Bozman, Senthil Vel Rajan Rajaram Manoharan, Tarak Vasavada

**Affiliations:** 1 Psychiatry, University of Alabama at Birmingham School of Medicine, Huntsville, USA; 2 Psychiatry, Huntsville Hospital, University of Alabama at Birmingham School of Medicine, Huntsville, USA

**Keywords:** sars-cov-2, antipsychotics, elderly, isolation, depression, cognitive decline, psychosis, alzheimer’s dementia, dementia, covid-19

## Abstract

Care for geriatric patients can be difficult due to the complex nature of age-related comorbidities, multiple medications, and cognitive decline; this hardship multiplies when psychiatric illness or dementia are present and often exacerbates existing issues. Millions of lives have been lost in the COVID pandemic, and it has also severely harmed our collective mental health and cognition. The elderly population has felt that this impact the greatest as they are at the highest risk of isolation, cognitive inactivity, loneliness, and depression, all of which are risk factors for dementia. Studies associate loneliness with a 40% increase in the risk of dementia; thus, this pandemic and resulting isolation have likely caused an increase in cognition loss of the elderly. Furthermore, there is a documented bidirectional relationship between COVID-19 and psychiatric illness, both of which increase the likelihood of the other and are associated with worsening mental cognition. We present a case series of two patients with pre-existing psychiatric illness and cognitive decline, both exacerbated by COVID-19 infection, causing further decline in cognition.

## Introduction

According to the World Health Organization, as of March 19, 2022, COVID-19 has infected 464 million people worldwide and taken the lives of six million people [1]. This pandemic has ebbed and flowed for several years now and, despite our best efforts, continues to affect our lives in meaningful ways. Much attention and resources have been devoted to understanding this virus; we have developed novel treatments and vaccines in record time. However, we know that immunocompromised patients, older patients, and those with numerous comorbidities are at the most significant risk of infection and death from COVID-19 [2]. This article presents a case series of patients already affected by chronic psychiatric illness with some baseline cognitive deficits who develop COVID-19 and the cognitive decline they face.

This trilemma of chronic psychosis with cognitive deficits, COVID-19, and the resulting damage-causing further cognitive decline is a scenario that is neither rare nor complicated. The CDC estimates that about 5.8 million people in the United States alone have Alzheimer’s and related dementias, with 5.6 million being older than age 65 [3]. Risk factors for dementia include diabetes, hypertension, obesity, depression, cognitive inactivity, and low educational attainment [4]. Psychosis is very common in persons with dementia; the incidence has been estimated to be 30% and can cause immense stress to caretakers [5]. There exists a large market of pharmaceuticals for the treatment of dementia, some of which aim to slow down the progression of the disease, and others are pharmaceutical cognitive enhancers, or nootropics, that improve cognition [5]. These drugs, such as antipsychotics, often have significant side effects and can also slow cognition in the elder. There is no cure for dementia. It is easy to understand how this pandemic and the isolation endured could be linked to increased rates of depression and cognitive inactivity. In fact, some studies associate loneliness with a 40% increased risk of dementia [6]. We present two case studies that illustrate a COVID-19 infection resulting in cognitive decline in those with some level of cognitive impairment associated with chronic psychosis.

## Case presentation

Case 1

The patient is a morbidly obese 65-year-old Caucasian female with a past medical history of recurrent pulmonary embolism, hyperlipidemia, and asthma with a prior inpatient hospitalization several years ago for psychosis in the elderly. Until recently, she was high functioning and was not reported by her family to have any current psychiatric diagnosis or baseline cognitive impairment. She presented to the Emergency Department (ED) for a psychiatric evaluation from the local sheriff’s office. She was brought in by her family with complaints of confusion and inability to complete activities of daily life. She had delusions of the death of her family and had been making remarks on social media that her sister had committed suicide. These claims were refuted by other family members, including her son. The family indicates that she had no delusions prior to this recent onset two weeks prior and that these delusions were not lingering effects from her prior episode of psychosis in the elderly. Her family stated that she has no history of substance use disorder. She had a family history of Alzheimer’s dementia and bipolar disorder but was not diagnosed with these conditions herself. She was found to have a UTI on the initial evaluation, which was treated promptly with levofloxacin and resolved without issue. While she was hospitalized in inpatient psychiatric care for the treatment of psychosis, she was found to be positive for COVID-19 on incidental testing 20 days after admission. She did not have any respiratory symptoms at that time. She was transferred to the medical floor for treatment. There, following the onset of COVID-19, she had a significant worsening in her sensorium, and she became further delirious, confused, and agitated. She was unwilling and unable to cooperate with any standardized cognitive assessment testing. Further neurological evaluation was done, including EEG and MRI. EEG showed diffuse slowing suggestive of diffuse cerebral dysfunction or diffuse encephalopathy. MRI showed mild atrophy with no appreciable disease, and other causes of delirium, such as vitamin deficiency, electrolyte imbalance, hypoglycemia, and infection were ruled out. She remained entirely confused, oriented only to name, and responding to internal stimuli for 31 days after testing positive for COVID during her total 51-day hospitalization despite the use of multiple antipsychotics, haloperidol and ziprasidone, due to continual extreme agitation and confusion and risk of harm to herself and staff. She was eventually discharged to a long-term care facility.

Case 2

The patient is a 65-year-old Caucasian female with a past medical history of hypertension, hyperlipidemia, myasthenia gravis, chronic obstructive pulmonary disorder on 2 liters of home oxygen, and a 30+ year history of medically stable paranoid schizophrenia who presented to the ED from a rehab facility due to recurrent falls. She did not require any cognition testing during this stay, which appeared to be her baseline. She had no family history of mental illness, and no history of substance use. She was medically stabilized over three weeks and discharged home only to return a day later due to aggressive and violent behaviors, and she then tested positive for COVID-19. She was mildly hypoxic but had no other respiratory symptoms. On this admission, she was delirious and incoherent, responding to internal stimuli and requiring soft physical restraints. She remained delirious and confused and began refusing to eat or take oral medications. She had evidence of cerebral atrophy on CT. She required the use of three antipsychotics, initially olanzapine and haloperidol, which were eventually tapered down and replaced with clozapine. Valproic acid was used as an adjunct therapy for extreme agitation and risk of harm to herself and staff during her stay. Antipsychotics were given to her mostly intramuscularly because of severe agitation and refusal to take oral pills. She had severe executive dysfunction, disinhibited behavior throughout her stay, and mainly perseverated on religion and on Jesus Christ. There was a subsequent decline in her functioning that accompanied poor oral intake. She required tube feeding and was briefly intubated due to respiratory collapse related to COVID-19. She had a prolonged recovery course but remained delirious, not being oriented to her situation and lacking any degree of insight, and only some improvement in agitation. She was eventually discharged to long-term nursing care as her family was unable to take care of her. This case again highlights the worsening cognition leading to delirium in a patient who had pre-existing chronic psychosis and mild cognitive impairment.

## Discussion

Special attention is required when caring for geriatric patients as they commonly have multiple comorbid conditions and are often treated with numerous medications. Care has been especially difficult during the COVID pandemic, as our elderly population was disproportionately affected. The mortality rate of COVID among the elderly was drastically higher globally. The SARS-CoV-2 virus has itself been linked to psychosis, particularly psychotic and delusional disorders, though the underlying pathogenesis is unclear [7]. The virus alone is not responsible for the vast increase in mental illness diagnoses in our elderly, as this pandemic has caused damage economically and physically as well. This includes increased rates of unemployment, homelessness, relationship breakdowns, domestic abuse, and social isolation [8]. Many countries took herculean efforts such as long lockdowns and aggressive isolation to shield their elderly populations from the virus [9]. Unfortunately, isolation, too, is associated with many physical and mental health issues, such as vascular and neurological diseases and premature mortality [10]. The aforementioned factors, and many more, show how complex and difficult it has been to balance public health and mental health during this pandemic. The patients we present above give specific examples of this difficulty, and how these mental and physical health conditions are interconnected.

The patients presented here had multiple medical comorbidities, chronic psychosis, and some level of baseline cognitive decline or dementia. Both cases exemplify the effect of COVID-19 on both chronic psychosis and cognitive decline. The patient in case 1, though delirious on admission, initially declined to participate in cognitive testing; however, following the contraction of COVID-19, there was a clear and drastic change in her sensorium and she became unable to participate in any level of cognitive assessment. The patient in case 2 had dealt with schizophrenia for many years and too had a rapid decline after contracting COVID-19. They had a precipitous drop in further cognitive functioning, and we portend that this drop is associated with infection of SARS-CoV-2 causing COVID-19. Unfortunately, due to the level of agitation and confusion, neither of the above patients was amenable to full cognitive evaluation, though bedside evaluation showed that severe attentional deficits were present and that their sensoriums were severely altered.

**Figure 1:**
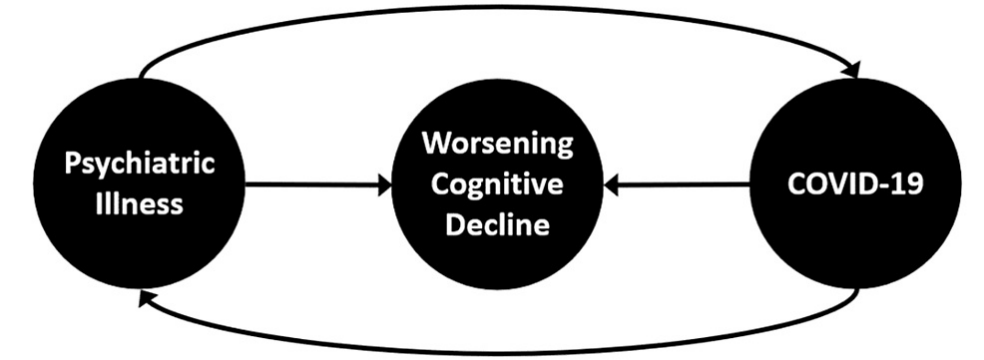
Example of the bidirectional relationship between psychiatric illness and COVID-19, both of which are associated with worsening cognitive decline.

SARS CoV-2 virus can directly access and cause damage to the neurons in addition to the cascade of stress responses and activation of pro-inflammatory cytokines [11]. A bidirectional relationship exists between COVID-19 and psychiatric illnesses, as demonstrated in Figure 1. Depression and dementia are well-recognized risk factors associated with increased rates of COVID-19 contraction, hospitalizations, and deaths [12]. Dementia and worsening functional status are associated with COVID-19; in fact, some have suggested that worsening dementia be considered an early sign of COVID-19 infection [13]. The incidence of psychiatric conditions is also increased in those infected with COVID-19 during the first 14-90 days at 18.1%, including 5.8% that was a first diagnosis [14]. Thus, psychiatric illnesses are correlated with increased rates of COVID-19 as well as COVID-19 is correlated with the onset of acute psychiatric illnesses, both of which are associated with worsening cognitive decline. Both our patients experienced rapid cognitive decline at the time of or shortly after developing a COVID-19 infection.

## Conclusions

Until the vaccination rate for all nations is high, there is no doubt that there will be more variants of the SARS-CoV-2 virus that causes COVID-19. We have learned many lessons during this time, but what remains clear is the vulnerability of the elderly and our need to address their mental health issues in conjunction with psychical health needs. Infection of this virus has health consequences on many organ systems in the human body, and it appears that mental cognition and a patient's general sensorium can suffer from a significant and long-lasting (if not permanent) loss. Dementia, delirium, and acute confusion are frustrating conditions that cause financial, mental, and emotional stress for patients and for family members, as well as the healthcare staff. It has been challenging to balance isolation to protect from the virus against the mental health damage incurred from this isolation. As seen from the mentioned cases, contracting COVID-19 can lead to precipitous and drastic confusion and delirium, making patient treatment and recovery difficult. We must assure that proper masking is maintained, as well as proper droplet and contact precautions to prevent viral transmission in the hospital setting. As more variants inevitably arise, it is of the utmost importance that we begin to understand and identify how COVID-19 affects cognition in the elderly to narrow our focus on who is at the highest risk. These are compelling reasons to increase mental health outreach before the next wave of infections.
